# Towards palliative care for all: Translation and cross-cultural adaptation of the Supportive and Palliative Care Indicators Tool-4ALL (SPICT-4ALL) to the Tamil healthcare context in India and Sri Lanka

**DOI:** 10.1017/S1478951526102442

**Published:** 2026-05-15

**Authors:** Shunmuga Priya I. C., Shamini Prathapan, Jyothsna Kuriakose, Gobith Ratnasingam, Ruby Angeline Pricilla S., Sajitha MF Rahman, Daniel Munday, Kirsty Boyd, Scott A. Murray, G.V.M.C. Fernando, Jenifer Jeba Sundararaj

**Affiliations:** 1 Department of Palliative Medicine, Christian Medical College, Vellore, India; 2 Department of Community Medicine, Faculty of Medical Sciences, University of Sri Jayewardenepura, Nugegoda, Sri Lanka; 3 Department of Medicine, University of Jaffna, Jaffna, Sri Lanka; 4 Department of Community Medicine, Christian Medical College, Vellore, India; 5 Department of Family Medicine, Christian Medical College, Vellore, India; 6 Primary Palliative Care Research Group, Usher Institute, University of Edinburgh, Edinburgh, UK; 7 Department of Palliative Care, University of St Andrews, St Andrews, UK; 8 Department of Primary Palliative Care, The University of Edinburgh, Edinburgh, UK; 9 Faculty of Graduate Studies, University of Sri Jayewardenepura, Nugegoda, Sri Lanka

**Keywords:** Supportive and Palliative Care Indicators Tool, Tamil, palliative care, translation, cross-cultural adaptation

## Abstract

**Objectives:**

We aimed to translate and cross-culturally adapt the Supportive and Palliative Care Indicators Tool-4ALL (SPICT-4ALL) for use in the Tamil healthcare context.

**Methods:**

The translation and cross-cultural adaptation of the SPICT-4ALL (2023 version) were conducted using the TRAPD model (Translation, Review, Adjudication, Pretesting, and Documentation). Cross-cultural adaptation used a modified Delphi-technique. Twelve participants, including mid-level healthcare workers from primary care and palliative care settings and lay people from India and Sri Lanka, scored the items on the tool. Agreement on scores was assessed, and focus group discussion (FGD) was used to reach consensus.

**Results:**

Delphi panel agreement was 34% initially but reached 100% with minor changes to items in the translated version after the FGD. Thematic analysis found SPICT-4ALL in Tamil is user-friendly and accessible for proactive identification of palliative care needs, facilitates person-centered care planning, and enhances interdisciplinary coordination.

**Significance of results:**

SPICT-4ALL-Tamil 2023 is the translated and cross-culturally adapted version for use in the Tamil healthcare context. This will enable and empower mid-level health workers within the primary and secondary care settings and people not in the healthcare field to identify individuals with palliative care needs. Further research to validate and study acceptability of the tool and its impact on patient outcomes is warranted.

## Introduction

Early palliative care integration in oncology and increasingly for other life-limiting illnesses has shown multiple benefits for patients, carers, and the healthcare system (Haun et al. 2017; Mós and Reis-Pina 2025). However, early identification of patients with palliative care needs remains a key challenge. WHO data from South East Asia suggest that 62% of deaths are due to noncommunicable diseases (NCDs) annually, accounting for a total 9 million (World Health Organization 2025). The majority (67.1%) of people requiring palliative care are adults over 50 years, and most of them (76%) live in low- and-middle-income countries (World Health Organization 2020). The Declaration of Astana, 2018, clearly states that palliative care is part of primary health care and needed to achieve Universal Health Care coverage (Declaration of Astana 2018).

Primary care physicians, nurses, and community health workers are best placed and suited to provide longitudinal care of individuals with NCDs including early identification and provision of primary palliative care. Primary palliative care is defined as palliative care practiced by primary healthcare workers, who are the principal healthcare providers for people in local communities throughout their lifespan. It includes early identification and triggering of palliative care as part of integrated and holistic chronic disease management, collaboration with specialist palliative care services (where available), and strengthening of underlying professional competencies in primary care (Munday et al. 2019).

Palliative care is integrated into the National Health Program of India (National Program for Palliative Care 2025). Similarly, in Sri Lanka, palliative care has been integrated into the national health system, first at the policy level in 2016 through the National Health Policy and further strengthened with the launch of the National Strategic Framework for Palliative Care Development in 2019 (National Program for Palliative Care 2025).

The Supportive and Palliative Care Indicators Tool (SPICT) (The SPICT I Supportive and Palliative Care Tool 2025) and Supportive and Palliative Care Indicators Tool for Lower Income Settings(SPICT-LIS) (The SPICT I Supportive and Palliative Care Tool 2025) are simple clinical tools that help identify individuals with deteriorating health who might benefit from a palliative care approach, alongside other forms of treatment or care. This tool is suited to busy clinical settings and covers most conditions encountered in primary care settings (Highet et al. 2014). These tools have been used and evaluated in different settings in high- and lower-income countries and can be used for all patients, in all contexts, and at any point in the illness trajectory (Boyd and Murray 2010; Highet et al. 2014; Gupta et al. 2024; Minato et al. 2024; Moraes and Azevedo 2024; ). The presence of medical terminology in SPICT or SPICT-LIS precludes its use by patients, family members, and mid-level healthcare workers.

To overcome this, SPICT-4ALL (2024) was developed by an English hospice staff working in local care homes with support from the SPICT program lead. SPICT-4ALL aims to make it easier for everyone, including patients and carers, to recognize and talk about signs that an individual’s overall health may be getting worse. It includes general health indicators and clinical indicators across various health conditions, along with a 5-item action plan written in nonmedical language. SPICT-4ALL has already been translated into German, Spanish, Portuguese, Danish, and Brazilian Portuguese (SPICT-4ALL 2024). A community survey in India successfully used SPICT-4ALL to identify palliative care needs in 2 rural communities (Sudhakaran et al. 2023).

Mid-level healthcare workers in countries like India and Sri Lanka play an essential role in healthcare delivery in institutional, residential, and community healthcare settings. In Tamil-speaking healthcare contexts within India and Sri Lanka (serving an estimated 80–90 million people) (Spread of the Tamil language 2025), it is important to equip and enable primary and mid-level health workers who predominantly use their native Tamil language to identify and support patients with palliative care needs. Therefore, this study aimed to translate and cross-culturally adapt SPICT-4ALL into Tamil primarily for use in the Tamil-speaking healthcare context. Availability of SPICT-4ALL in Tamil will enable primary care health workers to use it effectively, and promote better understanding among caregivers and patients about worsening health, unmet needs and how to receive more support.

## Methods

### Setting

**Figure 1.**
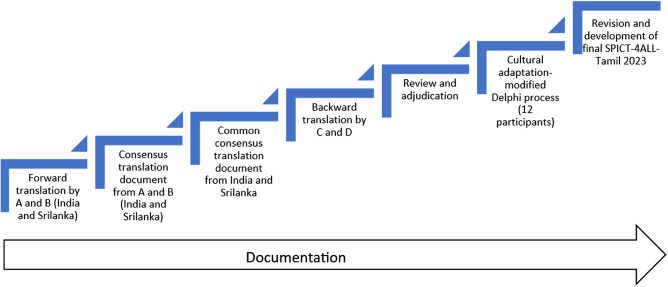
Steps in translation of SPICT-4ALL to Tamil.

The translation process of SPICT-4ALL (2023 version) was undertaken by a working group consisting of healthcare professionals from primary care and palliative care in Tamil Nadu, India, and Sri Lanka between May 2024 and February 2025. The TRAPD model (Translation, Review, Adjudication, Pretesting, and Documentation) (Harkness 2011) was used for the translation (Fig. 1). Translation was done adhering to the widely accepted forward–backward translation method, which has often been used for cross-cultural adaptation of health and care instruments (Kulis et al. 2017). Cross-cultural adaptation was done through a modified Delphi technique with 12 participants from Tamil Nadu, India, and Sri Lanka.

### Translation

#### Forward translation (English to Tamil)

Two healthcare professionals, A and B, whose mother tongue is Tamil, and who are fluent in English, translated the SPICT-4ALL to Tamil independently. During the translation process, the translators were asked to pay special attention to ensuring conceptual and normative equivalence rather than focusing merely on preserving the literal meaning of each word. They were asked to avoid technical terms and long sentences as much as possible, and encouraged to use simple and acceptable language to improve the understanding of target users. The research team, which had investigators fluent in both Tamil and English, compared the 2 forward Tamil translations with each other and the original English version. Discrepancies and ambiguities between the translated versions and the original English version were discussed by the principal investigator with translators A and B and new versions were created. This process was done independently in Tamil Nadu, India, and in Sri Lanka. Then, the 2 forward-translated Tamil versions were compared and discussed. A single, consensus-forward-translated version acceptable for both countries was created and used for backward translation.

### Backward translation (Tamil to English)

Two independent translators, C and D, who had Tamil as their mother tongue and were fluent in English, completely blinded to the original English version of SPICT-4ALL, translated the consensus-forward-translated version back into the English language. Following this, the 2 independent translators and the principal investigator compared the English translation with the original tool to determine any discrepancies or conceptual errors. This was addressed through consensus by the working group.

### Review and adjudication

All translations were reviewed to identify dissimilarities between translations. Words that did not match the original SPICT-4ALL tool were highlighted and given back to the English-language translators (C and D), and consensus was obtained following discussion. The translated Tamil version of SPICT-4ALL was finalized in the working group, and this version was used for the cross-cultural adaptation.

### Pretesting

A modified Delphi technique was used to test and adjust the translation so that the tool can be acceptable cross-culturally and adapted for healthcare situations in any Tamil-speaking context. This step of cross-cultural adaptation is necessary to overcome the limitations of a purely linguistic translation (Beaton et al. 2000). Twelve participants from India and Sri Lanka, with Tamil as mother tongue, who were healthcare staff working in primary care or palliative care in hospital or community settings, and individuals not involved in healthcare provision (lay people) were included to give a lay perspective and contributed to this process (Table 1). The participants were instructed to read the translated Tamil version of SPICT-4ALL and the English version carefully and voice any difficulties in meaning or understanding. The items in the SPICT-4ALL were numbered sequentially beginning with the title for facilitating the cross-cultural validation through the modified Delphi method. Each phrase numbered as item in the translated tool was checked for (1) Jargon (Is the most appropriate medical word that is culturally accepted used?), (2) Ambiguity (Is the text ambiguous or poorly worded?), (3) Multiple questions (Is 1 item asking for answers to 2 or more questions at the same time?), (4) Value-laden words (Are the words that prejudice the respondent, leading them to answer to the tone of the question?). The participants were asked to score each phrase on a scale of 1–5 (1. Hard to answer, 2. Confusing, 3. Hard to understand, 4. Disturbing/offensive, 5. No issues). The responses were analyzed, and the percentages of agreement for each item summarized after each round. Those items with an agreement of 100% were accepted. The items with agreement less than 100% were highlighted. Another round of Delphi was done, and items were modified based on group consensus.

**Table 1.** Demographic details of participants in the modified Delphi process

	Gender	Country	Designation/Role	Years of experience
S1	Male	Sri Lanka	Trainer	10
S2	Female	Sri Lanka	Manager^a^	9
S3	Male	Sri Lanka	Program officer	8
S4	Male	Sri Lanka	Allied health professional	20
S5	Female	Sri Lanka	Healthcare coordinator^a^	20
S6	Male	Sri Lanka	Social worker in a non-healthcare setting^a^	4
T1	Male	Tamil Nadu, India	Medical records officer^a^	2.5
T2	Female	Tamil Nadu, India	Staff nurse	27
T3	Male	Tamil Nadu, India	Community health worker	8
T4	Male	Tamil Nadu, India	Doctor	3
T5	Female	Tamil Nadu, India	Staff nurse	32
T6	Male	Tamil Nadu, India	Social worker	1

a Lay people.

The participants were also asked to answer the following open-ended questions to check for tool applicability.

1. How do you see SPICT-4ALL in Tamil being used and implemented in your clinical practice?
2. How long do you think that it takes to complete the checklist?

A thematic analysis following Braun and Clarke’s 6 phases was performed (Braun and Clarke 2006). This included an initial understanding of all the interviews, generating initial codes, searching for themes, and selecting appropriate extracts.

### Documentation

Throughout the translation process and cross-cultural adaptation of SPICT-4ALL (2023 version) in Tamil, every step undertaken was documented transparently. This can serve as a guide for future translations. The final Tamil version of SPICT-4ALL (SPICT-4ALL in Tamil 2023) is available for download on the SPICT international program website (SPICT-4ALL 2024).

#### Ethical considerations

The study was approved by the Institution Review Board and Ethics Committee of Christian Medical College Vellore (IRB Min No: 15953 dated 20.03.2024) and Research Ethics Committee of the Faculty of Medical Sciences at the University of Sri Jayewardenepura, Sri Lanka (Approval number: ERC 04/24). All study respondents received the participant information leaflet and verbal explanations of the process, and gave written consent.

## Results

The demographic details of the participants in the modified Delphi panel are summarized in Table 1. The majority were women with experience in healthcare work ranging from 1 to 32 years. Four participants were nonclinical staff and offered lay perspective. All of them were fluent in Tamil and English.

The Delphi panel achieved 100% agreement for 18 of 53 items in the translated version of SPICT-4ALL-Tamil. Of the 35 items which did not achieve consensus, 29 achieved 100% agreement after clarification and discussion during the focused group discussion (FGD) and were retained in their existing form. The remaining 6 items were modified till a consensus agreement (100%) was achieved regarding the acceptability of the phrase in Tamil and the relevance in the cultural context. The main reasons that led to the revisions were idiomatic equivalence (*n* = 2) and semantic equivalence (*n* = 4) (Table 2).

**Table 2.** Discrepancies identified according to the types of equivalences

Type of equivalence	Frequency	Item number
Idiomatic equivalence	*n* = 3	1, 10,^a^ 41
Semantic equivalence	*n* = 5	13, 16, 20,^a^ 24, 52
Experiential equivalence	*n* = 0	–
Conceptual equivalence	*n* = 0	–

a Not modified.

### Idiomatic equivalence

There is no equivalent Tamil translation of the word “palliative” that could be used directly in the name of the tool and in 1 of the general indicators “person (or family) asks for palliative care.” The English term “palliative” was therefore added in parentheses after the Tamil translated word with the closest meaning to palliative care. There are no direct Tamil translations for “Parkinson’s disease,” “multiple sclerosis,” or “motor neuron disease,” so the English names were retained in parentheses.

### Semantic equivalence

The phrase “less able to manage usual activities,” was amended to “lack of strength to perform daily tasks” to make its meaning clearer. The initial translation in Tamil for “short of breath when resting, moving or walking a few steps” was amended to add an “or” before moving. Use of the term “palliative care” in the phrase “choosing palliative care instead of starting dialysis” was discussed by the panel and agreed that the Tamil word used in the translation was appropriate for this context. The phrase “poor control of bladder and bowels” was amended to “loss of control of urination and defecation” to improve understanding in Tamil. The following phrase “We need to plan early if the person might not be able to decide things in the future” on translation, there was a lack of clarity so an additional word in Tamil was added to make it a continuous tense.

All 12 participants assessed the final Tamil version for jargon, ambiguity, multiple questions within an item, and value-laden words and reached full consensus agreement on the final translated version during an FGD. Key themes identified were “User-friendly and accessible tool for proactive identification of palliative care needs,” “Tool for facilitating person-centered care planning,” and “Tool for enhanced interdisciplinary coordination” (Table 3). Time for completion of a patient review with the tool as reported by the participants ranged from 10 to 30 min (average:15.3 min).

**Table 3.** Results of thematic analysis using Braun and Clarke’s 6 phases

Main theme	Subtheme	Coding	Examples of quotes from participants
User-friendly and accessible tool for proactive identification of palliative care needs	Usability and accessibility	Easy to use and integrate into the workflow	“used easily in any kind of setting” (T1,T6) “used easily and effectively in daily processes” (S4) “simple tool” (S1,S2,S5,T2)
	Proactive identification of palliative care needs	Enabling proactive identification of palliative care needs in any setting	“identify the needy patient like older age people, bedridden those who want special care (primary care)” (T3) “regardless of their setting, whether at home, in a care facility, or in a hospital” (T6) “used easily in any kind of setting” (T1,T6) “identifying patients with symptoms and those in need of palliative care” (S2,S3,S5) “to identify patients with palliative care needs earlier and more accurately” (T6)
Facilitates person-centered care planning	Comprehensive care planning	Facilitating person-centered care	“more comprehensive and person-centred care” (T2,T6) “potential to significantly improve our patient care by enabling the healthcare team to better comprehend and address their needs” (T6) “ensuring that patients receive the best possible support and management of their symptoms, pain, and distress” (T6) “ensure patients receive appropriate care as their health deteriorates, regardless of their diagnosis or stage of illness” (T4)
	Comprehensive care planning	Facilitate advance care planning	“… advance care planning, and avoid the burden of intense levels of care for those with a poor prognosis” (T4) “identification and discussion of declining health trends, enabling individuals, their loved ones, and caregivers to access timely support and care” (T6)
Promotes enhanced interdisciplinary coordination	Coordinated care and timely referral	Coordinated care to enable timely referrals	“facilitate communication between primary care providers, specialists, and palliative care teams to coordinate care effectively” (T4) “will facilitate timely referrals to specialist palliative care services” (T6)

The final SPICT-4ALL in Tamil 2023 is published on the SPICT website (https://www.spict.org.uk/spict-4all/india-and-sri-lanka/) and available for use.

## Discussion

This research study is the first to translate and cross-culturally adapt the SPICT-4ALL tool as a plain-language version of the SPICT in Tamil. This is also the first published research on translation to 1 of the main “Indian” languages. It marks an important step toward strengthening primary palliative care services in Tamil Nadu, South India, and Sri Lanka, a region with growing palliative care needs but limited specialist resources. This work is unique as it marks collaboration across countries (India and Sri Lanka) and across disciplines (palliative care and primary care) and a multidisciplinary working group with lay people in it.

Our findings showed that approximately 66% (35 out of 53) of the phrases in the translated Tamil version presented some degree of ambiguity. This highlighted the sociocultural and linguistic heterogeneity that characterizes health-related communication across different regions and dialects in Tamil-speaking populations. They also underscored the critical importance of conducting rigorous cross-cultural adaptation, rather than relying solely on direct translation methods, before integrating such tools into routine clinical practice.

The use of the TRAPD methodology, which is endorsed by the Guidelines for Best Practice in Cross-Cultural Surveys (Harkness 2011), ensured a structured, iterative, and culturally sensitive approach to the translation process. This methodology, which has previously been employed in the Danish and German adaptations of the SPICT tool (Afshar et al. 2018; Bergenholtz et al. 2022; The SPICT I Supportive and Palliative Care Tool 2025), was facilitated by the involvement of a multidisciplinary expert panel and allowed for thorough consensus-building at each stage. Although time-intensive, the TRAPD approach enhanced both the cultural relevance and linguistic clarity of the Tamil version within India and Sri Lanka and ensured transparency and reproducibility through meticulous documentation.

The expert multidisciplinary panel for this study included palliative care physicians, primary care doctors, nurses, and social workers. This diverse membership enriched the translation process by integrating varied professional perspectives on how palliative care needs are expressed and recognized in clinical and community contexts (Oishi et al. 2022). Inclusion of staff able to provide robust lay perspectives during the pretesting phase further strengthened community-level applicability of SPICT-4ALL in Tamil. Their involvement improved the simplicity of language chosen, reduced residual medical jargon, and ensured greater acceptability and usability for nonspecialist settings.

This is the first example of a collaborative project to translate SPICT-4ALL by clinical research teams from 2 countries with a common language, making the translation and cross-cultural adaptation process and the planned use of the tool an important innovation. Though Tamil is a language spoken in the state of Tamil Nadu in India and in Sri Lanka, some words still remained unique to each country. This posed an additional challenge addressed by paying close attention to nuanced meanings and allowing for more discussions on the choice of words acceptable for use in both countries. The networking between palliative and primary care physicians in this study has served to drive forward new interest that can advance palliative care across 2 different healthcare systems.

The study working group initially came together with the intention of translating the standard SPICT to Tamil. However, most or all healthcare professionals in both countries speak and understand English. The group identified a greater need for a translation of SPICT-4ALL. That will allow mid-level health workers in different healthcare settings to use it, contribute to early identification of individuals with palliative care needs, and promote a palliative care approach in their workplaces. In resource-constrained settings like India and Sri Lanka and other low- and lower-middle-income areas, there is a pressing need for simple, time-efficient, and culturally appropriate tools to aid in the timely identification of individuals with palliative care needs.

Key themes from the FGD confirmed that SPICT-4ALL in Tamil 2023 was simple, user friendly to identify individuals with palliative care needs, and able to promote comprehensive person-centered care. This includes offering advance care planning and making timely referrals in primary care within Tamil healthcare settings in India and Sri Lanka. Hence, this linguistically validated and contextually adapted tool will empower primary care providers in initiating timely supportive interventions, mobilizing relevant services, and developing individualized care plans.

However, while this study provides a strong foundation for the use of this tool in primary care settings, developing relevant training on its use and further research on its implementation in care are warranted. Psychometric validation studies are needed to assess the tool’s reliability, validity, and clinical utility in real-world practice. Future work should also explore its impact on care outcomes and its acceptability among community health workers, patients, and families.

## Conclusion

SPICT-4ALL is translated and cross-culturally adapted for use in Tamil healthcare contexts. The clear methodology documented provides guidance for future translation projects. Availability of SPICT-4ALL in Tamil for use in primary and community palliative care settings within Tamil healthcare contexts, especially by mid-level health workers, will facilitate early identification of patients who would benefit with a palliative care approach alongside other appropriate treatment. Training on its use will enhance confidence in the use of this tool to support palliative care provision. Further research on acceptability of the tool among end-users and impact of its use on patient outcomes are needed.
